# Investigation of Climate Effects on the Physiological Parameters of Dairy Livestock (Cow vs. Buffalo)

**DOI:** 10.3390/s24041164

**Published:** 2024-02-10

**Authors:** Nadia Piscopo, Roberta Matera, Alessio Cotticelli, Lucia Trapanese, Oscar Tamburis, Roberta Cimmino, Angela Salzano

**Affiliations:** 1 Department of Veterinary Medicine and Animal Production, Federico II University, 80137 Naples, Italy; nadia.piscopo@unina.it (N.P.); roberta.matera@unina.it (R.M.); alessio.cotticelli@unina.it (A.C.); angela.salzano@unina.it (A.S.); 2 Department of Electrical Engineering and Information Technologies, Federico II University, 80125 Naples, Italy; 3 Institute of Biostructures and Bioimaging National Research Council, 80145 Naples, Italy; oscar.tamburis@ibb.cnr.it; 4 Associazione Nazionale Allevatori Specie Bufalina (ANASB), 81100 Caserta, Italy; r.cimmino@anasb.it

**Keywords:** temperature–humidity index, milk production, precision livestock farming, sensors, dairy

## Abstract

Nowadays climate change is affecting the planet’s biodiversity, and livestock practices must adapt themselves to improve production without affecting animal welfare. This work investigates the influence that some climatic parameters such as Environment Temperature, Relative Humidity, Thermal excursion and Temperature–Humidity Index (THI), can have on milk quantity and quality in two different dairy species (buffaloes and cows) raised on the same farm. A further aim was to understand if THI threshold used for cows could also be used for buffaloes. The climatic parameters were recorded daily through a meteorological station located inside the farm. Milk quantity (converted into ECM) and quality (Fat Percentage—FP; Protein Percentage—PP; Somatic Cell Count—SCC) were measured. Data were analyzed with Spearman’s correlation index, separately for buffaloes and cows. The results indicate a greater sensitivity of cows to heat stress and a strong negative correlation of the ECM with meteorological data (*p* < 0.01). The results of this study may stimulate the use of integrated technologies (sensors, software) in the dairy sector, since the IoT (sensors, software) helps to enhance animal well-being and to optimize process costs, with a precision livestock farming approach.

## 1. Introduction

Nowadays, Precision Livestock Farming (PLF) is called to cope with the many issues related to sustainability of productions, since the food demand is growing together with human population. An improvement in food demand should be achieved without affecting animal welfare and the eco-sustainability of the farm. To achieve these goals, PLF has adopted various technologies, such as electronic bins, automated milking systems and monitoring devices. Among all available technologies, the use of Internet-of-things (IoT)-based systems is very widespread to monitor animals and, in general, barn condition [1]. Nowadays, much of the literature has described the successful employment of connected sensors in the dairy sector. For example, the IoT can be used for detecting specific diseases [2], ensuring the correct amount of nutrients [3] or controlling the environmental parameters to assess animal health and production [4]. Among all the aspects that the IoT allows us to monitor, one of the most important is animal well-being. There are several studies on the stressogenic factors affecting different animal species. Cortisol has been used in domestic [5,6,7] and wild [8,9] animals to measure stress levels and to evaluate the relative state of well-being/discomfort in relation to different environmental stimuli or the different farming and transport conditions of the animals. In recent years, due to the well-established phenomenon of climate change, data relating to meteorological and climatic conditions have been analyzed by studying the influence that heat stress can causes on animal reproduction, production performance and animal welfare [10,11,12]. The dairy industry is one of the most vulnerable to global warming and climate change, since dairy animals are more sensitive to heat stress due to the metabolic heat load produced by both animal digestion and milk synthesis [10,13]. In this regard, the temperature–humidity index (THI), as a bioclimatic parameter, combines the effect of air temperature and humidity and it is commonly used to study heat stress in dairy farms [14,15]. Many authors have evaluated the effects of THI and heat stress in dairy cattle, showing lower milk production and changes in milk composition with increasing THI levels [16,17]. Few studies, however, have evaluated the effect of heat stress on buffaloes, especially in buffaloes raised under intensive conditions and at Italian latitudes [15,18]. Although buffaloes are known to be able to adapt perfectly to different environments, they exhibit signs of great discomfort when exposed to direct solar radiation. The body temperature of buffaloes is slightly lower than that of dairy cattle, and their skin is generally black and poorly protected by hair, and thus more prone to absorbing heat [19]. In addition, buffalo skin has a density of sweat glands six times lower than cattle, a characteristic that makes heat dissipation by sweating inefficient [20].

Little information is available on the effect of THI in Italy in the light of new scientific information present. Hence, this work was carried out to perform sustainable, sensor-based measures to outline the THI thresholds at which environmental stress (heat or cold) arises in dairy buffaloes through the evaluation of milk quantity and quality characteristics. These intervals were compared with those obtained from dairy cattle raised under the same environmental conditions. This study aims, therefore, to show that the timely deployment of smart farming systems may deliver improvements in animal welfare. This will, in turn, enhance sustainable environmental technology for cleaner and greener growth [14,21].

## 2. Materials and Methods

### 2.1. Experimental Design and Selection of Animals

In order to limit the influence of climate variability on the sample, the experimental scheme was structured within a single dairy farm in Southern Italy, located in the municipality of Baia e Latina in the province of Caserta, which raised both buffaloes and dairy cows. To cover the entire period of twelve months, data collection was carried out in the period of March 2022–February 2023.

The comparison between the two animal species was possible due to the availability of twenty Italian Mediterranean buffaloes (3.5 ± 0.6 parity) and twenty Holstein dairy cattle (3.6 ± 0.2 parity), kept in stable conditions and milked twice a day.

Both buffaloes and cows were housed in free stall barns with concrete floors, independent and close together. During the study, a space availability of 15 m^2^/head and 80 cm of front feeder was guaranteed. Straw was used for bedding, which was renewed every two days. The information relating to each individual was officially provided by the Italian Breeders Association and included the following: animal ID, date of birth, date of calving, order of calving, Days in Milk (DIM), daily milk production (Milk), Fat Percentage (FP), Protein Percentage (PP) and Somatic Cell Count (SCC).

### 2.2. Analysis Processing and Dataset

Milk quality (fat and protein) was assessed through mid-infrared spectroscopy (Milkoscan FT6000^®^, Foss Electric, Hillerơd, Denmark). The SCC was analyzed by Fossomatic FC^®^ counter (Foss, Hillerơd, Denmark). For the measurement and logging of weather data, an IoT platform has been used. It was composed of a solar-powered weather station and a cloud platform for its management, data viewing and recording. The main specification of the weather station were reported in Table 1.

The information relating to environmental temperature (ET), relative humidity (RT), air pressure, wind speed and direction, as well as precipitation and ultraviolet radiation were recorded from March 2022 to February 2023 with the Wireless Transfer System every day at an hourly interval and stored on the breeder’s computer. From ET and RH collected each hour were obtained ET mean (ET_mean), ET minimum (ET_min), ET maximum (ET_max), RH mean (RH_mean), RH minimum (RH_min) and RH maximum (RH_max), TE (Thermal Excursion). Temperature–humidity index (THI) was calculated according to the literature, as follows [15,21]:

THI_ijk_ = (1.8 × ET_ijk_ + 32) − (0.55 − 0.55 × RH_ijk_) × (1.8 × ET_ijk_ + 32) − 58 (1)

The subscripts ijk mean: i = mean, j = min and k = max. The subscript denoted that the same equation was true for computing the THI_ mean (i), THI_min(j) and THI_max(k) adjusted with the proper ET and RT.

Original dataset included 390 records for 14 numeric variables. The features regarded the milk yield (Milk), quality parameters (FP, PP and SCC) and the monthly minimum, mean and maximum of temperature, humidity, THI and monthly values of thermic excursion. The milk quantity was turned in Energy Correct Milk (ECM) through the equation [22]:

*ECM* = L × (1 + ((X − 4) + (Z − 3.1)) × 0.1155 (2)

*L*, *X* and *Z* represented the amount of milk (in kg) and its fat and protein content (%).

In addition, new categorical variables were added to evaluate the influence of ET_mean and THI_mean on the milk yield and quality for both species.

### 2.3. Statistical Analysis

Spearman’s correlation analysis was carried out on the entire dataset, and on other two subsets based on ET and THI outset. The empirical threshold for ET_mean (ET_mean > 27 °C) was set to study the correlation of ECM, FP, PP and SCC at higher temperatures. This threshold was obtained from a mean of the ET_mean of summer months. For THI, a threshold of 72 [15] was chosen to analyze the correlation of milk yield and quality when the animals were under heat stress conditions. The statistical analysis was performed on the R software, version 4.2.2. The function “rcorr” from the “Hmisc” package was used for computing the correlation, while for the representation the function “corrplot” from package “corrplot” was employed. Moreover, functions from “dplyr” package were employed for data cleaning and organize. A *p*-value < 0.05 was considered statistically significant.

## 3. Results and Discussion

Table 2 showed the mean ± s.e of the ECM and quality parameters of cows and buffalo during experimental period.

The results of the monthly monitoring of the breeding environmental conditions are reported in Table 3.

**Figure 1:**
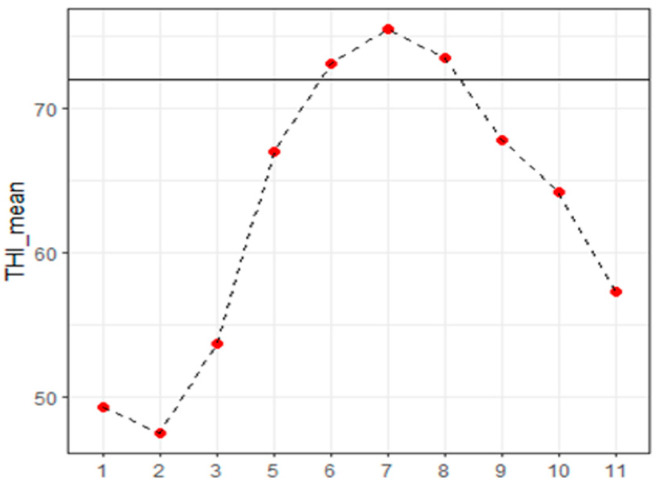
Trend of THI for each month recorded by the weather station of the farm. The black line represented the heat stress threshold.

The breeding area was characterized by a mild climate during the whole year. Indeed, only in June, July and August the THI_mean slightly exceeded its heat stress threshold (73.1, 75.5 and 73.5 vs. THI_mean = 72) [23]. This evidence is highlighted in Figure 1, which describes the trend of THI during the experimental period. This means that animals did not suffer much heat stress regardless of the time of the year. However, despite the warm climate, the analysis performed showed that the weather data influenced milk yield and quality confirming previous results in the literature [23].

**Figure 2:**
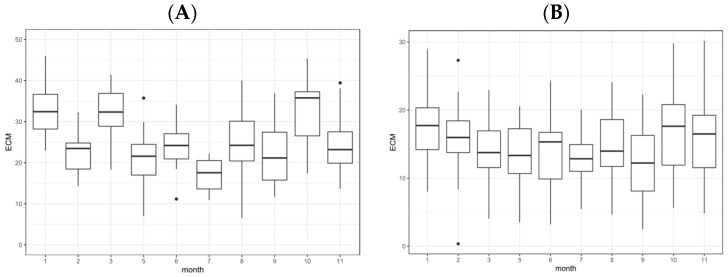
(**A**) Monthly trend of ECM for cows. Graphs refer to only the months in which ECM sample were available for cows. (**B**) Monthly trend of ECM for buffalo. Graphs refer to only the months in which ECM sample were available for buffalo.

The trend over the months is shown in Figure 2A,B for cows and buffaloes, respectively. It is possible to note a general decrease of ECM during the hottest months (6, 7, 8) for both species. However, as confirmed in the literature [14,23], for cows, the loss in milk production is more evident compared to buffalo.

### 3.1. Spearman Correlation on Buffaloes’ Data

**Figure 3:**
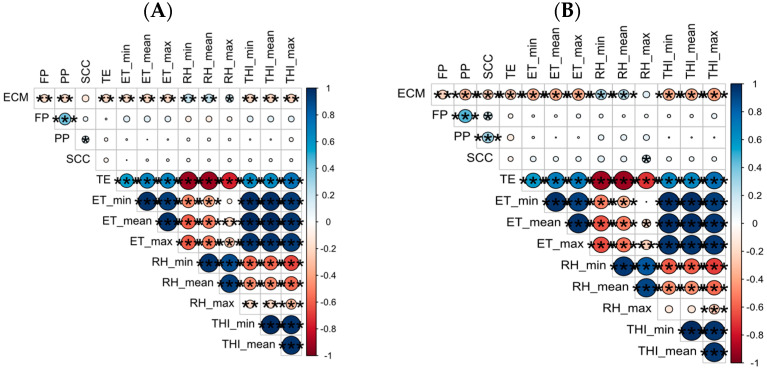
(**A**) General correlation matrix of buffalo performances without thresholds. (**B**) General correlation matrix of cow performances without thresholds. The size of circles represents the magnitude of correlation coefficients. ECM: Energy Corrected Milk; FP: Fat Percentage; PP: Protein Percentage; SCC: Somatic Cell Counts; TE: Thermal Excursion; ET: Environmental Temperature; RH: Relative Humidity; THI: Temperature–Humidity Index. Stars define the statistical significance of the correlation. In particular: * *p* < 0.05; ** *p* < 0.01; *** *p* < 0.001.

Spearman’s correlation analysis on buffalo data (Figure 3A) showed that ECM had significative correlations with the meteorological data. In particular, ECM had low negative correlations with ET_min, ET_mean, ET_max and THI_min, THI_mean and THI_max, (rho: −0.18, −0.21, −0.20, −0.20, −0.20, −0.21, respectively; *p* < 0.01). Low positive correlations were found among ECM and RH_min, RH_mean and RH_max (rho: 0.17, 0.21, 0.22, respectively; *p* < 0.01 and for RH_max *p* < 0.05). No significative correlations were found for FP, PP and SCC and environmental data.

### 3.2. Spearman’s Correlation on the Cow Data

Figure 3B showed the correlation matrix for Holstein cows. Spearman’s correlation analysis showed that ECM had significative correlation with the meteorological data. The analysis performed on the cow data showed that the ECM had strong, significant negative correlation with ET_min, ET_mean, ET_max and THI_min, THI_mean and THI_max, (rho: −0.35, −0.35, −0.36, −0.35, −0.35, −0.41, respectively; *p* < 0.001). Milk quality parameters had no significative correlations, except for with SCC which showed a low positive correlation with RH_max (rho: 0.16, *p* < 0.05).

### 3.3. Effects of High Temperatures on Buffaloes and Cows

When the ET_mean exceeded the 27 °C, the Spearman’s correlation analysis for buffaloes did not return any significative correlation, However, it was possible to denote a trend. Indeed, the ECM was negatively correlated with ET_min, ET_mean, ET_max, THI_min, THI_mean and THI_max, RH_max and RH_mean (rho: −0.05, −0.04, −0.05, −0.02, −0.04, −0.03, −0.04, −0.05, respectively; *p* > 0.05). A low positive trend was found between ECM and RH_min which showed no significant differences. The majority of the literature has focused on addressing the issue of heat stress in buffaloes raised in regions characterized by a tropical and/or sub-tropical climate [24,25]. However, there is a limited number of studies examining the impact on Mediterranean buffalo breeds in temperate climates. Most researchers [15,26] did not observe any effects of the hot season on MY in Mediterranean Italian buffalo. This divergence may be attributed to the buffalo’s enhanced adaptability to high temperatures [26]. On the contrary, it seems that the Mediterranean buffalo breed is more susceptible to low temperatures [26]. Matera et al. [15] noted an adverse impact on Milk Yield (MY) when the THI fell below 59, indicating that lower temperatures and humidity might negatively affect MY. However, in our study there was no effect of cold temperature on milk production.

It has been also seen [27] that, in terms of milk production, Mediterranean Italian buffalo exhibited a decline in milk quality under hot conditions, while the impact on milk yield was minimal. Conversely, in our study, milk quality was not affected by environmental conditions. Among buffalo breeds is the Murrah, which experienced the most significant reduction in milk production when subjected to heat-stress conditions, and not the Mediterranean buffalo [26].

**Figure 4:**
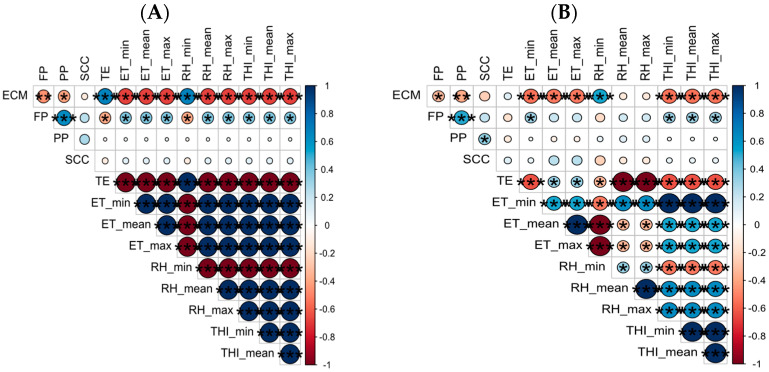
(**A**) Correlation matrix of cow performances when the ET_mean was >27 °C. (**B**) Correlation matrix of cow performances when THI threshold was >72. The size of circles represents the magnitude of correlation coefficients. ECM: Energy Corrected Milk; FP: Fat Percentage; PP: Protein Percentage; SCC: Somatic Cell Counts TE: Thermal Excursion; ET: Environmental Temperature; RH: Relative Humidity; THI: Temperature–humidity Index. Stars define the statistical significance of the correlation. In particular: * *p* < 0.05; ** *p* < 0.01; *** *p* < 0.001.

The analysis carried out on Holstein cows (Figure 4A) returned strong negative correlations between ECM and ET_min, ET_mean, ET_max (rho: −0.67, −0.66, −0.66, respectively; *p* < 0.0001). Also, the THI_min, THI_mean and THI_max had the same behaviour with ECM (rho: −0.67, −0.65, −0.66, respectively; *p* < 0.0001).

In addition, strong negative correlations among ECM, RH_mean and RH_max (r: −0.66, −0.67, respectively; *p* < 0.0001) were found. Moreover, RH_min had a strong positive correlation with ECM (r: 0.65; *p* < 0.001). These results are in agreement with the literature [28] and confirmed the negative role of heat stress in milk production in cows.

The FP, indeed, had positive correlations with the ET_min, ET_mean, ET_max, THI_min, THI_mean, THI_max, RH_mean and RH_max (rho: 0.35, 0.36, 0.34, 0.37, 0.36, 0.39, 0.37, 0.37, 0.37, respectively; *p* < 0.05) and negative ones with TE and RH_min (rho: −0.37, −0.37; *p* < 0.05).

### 3.4. Effects of Heat Stress on Buffaloes and Cows

When the THI_mean was higher than 72, no significant correlations were found for buffaloes. Indeed, it was possible to denote the same trend of the analysis performed when the temperature exceeded the threshold of 27 °C. The ECM was negatively correlated with ET_min, ET_mean, ET_max, THI_min, THI_mean and THI_max, but the correlations did not reach significance (rho: −0.10, −0.11, −0.09, −0.02, −0.02, −0.04, respectively; *p* > 0.05). However, in this case, RH_min, RH_mean and RH_max were positively correlated with ECM (rho: 0.10, 0.07, 0.08, respectively; *p* > 0.05). The FP showed the opposite behaviour. Indeed, among FP and ET_min, ET_mean, ET_max, THI_min, THI_mean and THI_max low, no significant positive correlations (rho: 0.08, 0.19, 0.20, 0.08, 0.09, 0.10, respectively; *p* > 0.05) have been found. The RH_min, RH_mean and RH_max were negatively correlated with FP (rho: −0.19, −0.09, −0.09; *p* > 0.05).

The analysis performed on Holstein cows (Figure 4B) showed that there were moderate significant negative correlations between ECM and ET_min, ET_mean, ET_max, THI_min, THI_mean and THI_max (rho: −0.54, −0.52, −0.52, −0.54, −0.53 and −0.54, respectively; *p* < 0.0001). The RH_min had a positive correlation with ECM (rho: 0.54; *p* < 0.001). On the other hand, the FP had positive significative correlations with ET_min, THI_min, THI_mean and THI_max (rho: 0.31, 0.30, 0.31, 0.32, *p* < 0.05).

In summary, both ET and THI influenced more the production performances of cows, rather than buffaloes. Regarding buffaloes, generally the high temperature (ET_mean > 27 °C) and a THI greater than 72 only led to a lower (but not significant) decrease of milk yield. Holstein cows showed the same trend but with strongest correlation coefficients. The ECM for Holstein was positively correlated with RH and negatively correlated with ET and THI. It is important to denote that the weather data had more influence on ECM and FP than PP and SCC. Indeed, out of all cases examined, fewer significant correlations were found for these variables. Moreover, the correlation coefficient for PP and SCC were generally lower compared to ECM and FP. Taken together, our results suggested a closer link within ECM and meteorological conditions for both species, in line with the scientific literature. Moreover, the effect of THI and high temperatures on buffalo differed from those observed in dairy cattle, hence the use of a same threshold would be not appropriate. In the context of “Smart Farming”, together with the weather station, further devices, able to interact with each other, should be employed. Only in this way would breeders be able to obtain all the information useful to manage, in an all-round, automatic way, a farm (e.g., cooling systems and heaters can be turned on and off automatically without human labor). Moreover, since heat stress can be shown in different way by animals, the monitoring of behaviour could be very precious information. In this context, a lot of devices are available on the market (collars and pedometers) that may interact with different kinds of IoT platforms [29,30] through a more integrated system.

## 4. Conclusions

The results of this study demonstrated that the use of sensors and software dedicated to data collection linked to the daily activities of dairy livestock may help to understand the different responses of the species to climatic variables. The analysis of our data confirmed what is reported in the literature for dairy cows. Indeed, cattle appear to be more affected by heat stress compared to buffaloes. Conversely, probably due to its tropical origin, the buffalo species appears to be less affected by heat stress.

It appears undeniable, however, that rapid climate changes have, and will have, an increasingly greater influence on the life of farmed animals and their production. It will therefore be necessary to increase observations in different environmental conditions and increase studies to evaluate and validate new levels of THI thresholds to be used specifically for buffaloes. The dairy sector has been asked to reduce the resources necessary for production and increase the sustainability of related activities. To this regard, the IoT with integrated technologies (sensors, actuators and software) is the most current support that also allows measurements of sustainability to be achieved.

## Figures and Tables

**Table 1:** Specification of all features measured by the weather station.

Features	Unit	Range	Accuracy	Resolution
Temperature	°C	−40–60	±1	0.1
Relative Humidity	%	10~99	±5	1
Rain volume	mm	0–6000	±10	1
Wind Speed	m/s	0–50	±1 (<5 m/s) or ±10% (>5 m/s)	-
Light	Lux	0–200	±15%	-
Pressure	hPa	700–1000	±3	0.1

**Table 2:** ECM: Energy Correct Milk; FP: Fat Percentage; PP: Protein Percentage; and SCC: Somatic Cell Count (Mean ± s.e.).

Variable	Cow	Buffalo
ECM (Kg)	25.7 ± 0.6	14.8 ± 0.4
FP (%)	3.9 ± 0.08	8.3 ± 0.1
PP (%)	3.4 ± 0.03	4.6 ± 0.02
SCC cells × 10^3^	153.5 ± 14.4	196 ± 12.0

Data are shown as mean ± s.e. ECM: Energy Correct Milk; FP: Fat Percentage; PP: Protein Percentage; and SCC: Somatic Cell Count.

**Table 3:** Summary of meteorological data recorded in the experimental dairy farm.

Month	TE_Mean (°C)	ET_Mean (°C)	RH_Mean (%)	THI_Mean
March/2022	15.2 ± 1,2	11.3 ± 0.5	48.0 ± 1.9	53.7 ± 0.6
April/2022	15.0 ± 0.9	14.9 ± 0.5	53.7 ± 2.0	58.1 ± 0.7
May/2022	16.2 ± 0.7	21.7 ± 0.6	51.6 ± 1.5	67.0 ± 0.7
June/2022	17.2 ± 0.7	27.1 ± 0.3	45.8 ± 1.0	73.1 ± 0.4
July/2022	16.4 ± 0.4	28.6 ± 0.2	47.6 ± 1.6	75.5 ± 0.4
August/2022	15.2 ± 0.4	26.0 ± 0.3	57.2 ± 1.6	73.5 ± 0.4
September/2022	11.1 ± 0.9	21.8 ± 0.8	63.0 ± 2.1	67.8 ± 0.9
October/2022	15.0 ± 0.6	18.9 ± 0.2	64.0 ± 0.9	64.2 ± 0.3
November/2022	9.72 ± 0.8	14.0 ± 0.5	68.4 ± 1.3	57.3 ± 0.8
December/2022	9.34 ± 1.7	12.6 ± 0.4	76.6 ± 0.9	55.1 ± 0.6
January/2023	9.14 ± 1.5	8.8 ± 0.4	71.6 ± 1.5	49.3 ± 0.7
February/2023	13.2 ± 1.4	6.8 ± 0.5	54.3 ± 1.9	47.5 ± 0.7

Data are shown as mean ± s.e. TE: Thermal Excursion; ET: Environmental Temperature; RH: Relative Humidity; and THI: Temperature–humidity Index.

## Data Availability

The data that support the findings of this study are available from the corresponding author upon reasonable request.
